# Effect of radiotherapy on survival in advanced hepatocellular carcinoma patients treated with sorafenib: a nationwide cancer-registry-based study

**DOI:** 10.1038/s41598-021-81176-w

**Published:** 2021-01-15

**Authors:** Shou-Sheng Chu, Yu-Hsuan Kuo, Wen-Shan Liu, Shih-Chang Wang, Chung-Han Ho, Yi-Chen Chen, Ching-Chieh Yang, Hung-Chang Wu

**Affiliations:** 1 Department of Radiation Oncology, Chi Mei Medical Center, No. 901 Zhonghua Rd., Yung Kang District, Tainan City, 701 Taiwan, ROC; 2 Department of Hematology and Oncology, Chi Mei Medical Center, No. 901 Zhonghua Rd., Yung Kang District, Tainan City, 701 Taiwan, ROC; 3 Department of Cosmetic Science, Chia-Nan University of Pharmacy and Science, Tainan, Taiwan, ROC; 4 Department of Radiation Oncology, Kaohsiung Veterans General Hospital, Kaohsiung, Taiwan, ROC; 5 Department of Nursing, Meiho University, Pingtung, Taiwan, ROC; 6 School of Medicine, National Defense Medical Center, Taipei, Taiwan, ROC; 7 Department of Radiation Oncology, Antai Medical Care Corporation Antai Tian-Sheng Memorial Hospital, Pingtung, Taiwan, ROC; 8 Department of Medical Research, Chi Mei Medical Center, Tainan, Taiwan, ROC; 9 Department of Hospital and Health Care Administration, Chia Nan University of Pharmacy and Science, Tainan, Taiwan, ROC; 10 Department of Pharmacy, Chia-Nan University of Pharmacy and Science, Tainan, Taiwan, ROC

**Keywords:** Oncology, Cancer, Hepatocellular carcinoma

## Abstract

Sorafenib is the standard treatment for advanced hepatocellular carcinoma (HCC) patients. This study aims to determine whether combining radiotherapy with sorafenib administration increases its efficacy. The study cohort included 4763 patients with diagnosed advanced HCC who received sorafenib between January 2012 and December 2015, as reported in medical records in the Taiwan Cancer Registry database. The effect of sorafenib with or without radiotherapy on survival was calculated using the Kaplan–Meier method and compared using the log-rank test. A Cox proportional hazards model was used for multivariate analysis. Patients receiving sorafenib plus radiotherapy had greater 1-year survival than did those receiving sorafenib alone (*P* < 0.001). Uni- and multivariate analyses also showed that radiotherapy increased survival after adjusting for confounders (adjusted HR 0.57; 95% CI 0.51–0.63). Further stratified analysis according to the timing of radiotherapy relative to sorafenib treatment revealed that patients who underwent radiotherapy after sorafenib had greater 1-year survival than did those undergoing radiotherapy within sorafenib use or sorafenib alone (adjusted HR 0.39; 95% CI 0.27–0.54). Combined treatment with sorafenib and radiotherapy results in greater HCC patient survival and should be considered an option for treating this challenging disease.

## Introduction

Hepatocellular carcinoma (HCC) is the third leading cause of cancer deaths worldwide^1^. The overall 5-year survival rate is only 5%, in part because 70% of patients are diagnosed with advanced stage disease, which has limited treatment options^2^. Sorafenib, a multi-kinase inhibitor against tumor proliferation and angiogenesis, is the first proven molecular targeting agent and the recommended standard therapy for advanced HCC^3,4^. However, two randomized placebo-controlled phase III trials have shown that monotherapy with sorafenib provides a low response rate and marginal survival benefit of less than 3 months^5,6^. Therefore, combining sorafenib with other therapies to improve outcomes is under active investigation^7,8^. The use of sorafenib with radiotherapy shows encouraging results with respect to patient response and survival rates^9,10^.

Previous studies have investigated the feasibility and efficacy of radiotherapy combined with sorafenib for treating advanced HCC^11,12^. Cha et al. report a notable tumor response and acceptable toxicity profile^11^. In a retrospective study, Wada et al. demonstrated that this combined modality is a suitable treatment for patients with extrahepatic spread and macrovascular invasion^13^. Preclinical data also indicate that the action of sorafenib as a VEGF inhibitor may have a radiosensitizing effect^14^. Although encouraging, these studies were generally limited by a small sample size, and few investigated the timing of radiotherapy intervention. Wild et al. observed greater efficacy in sequential rather than concurrent sorafenib and radiotherapy, both in vitro and in vivo^15^*.*However, inconsistent findings were reported by another study^16^. Therefore, further investigation is needed to explore this correlation.

This study aims to assess the effect of radiotherapy and its timing on survival in advanced HCC patients receiving sorafenib. The patient cohort was selected from the nationwide cancer registry database in Taiwan, which includes complete information regarding sorafenib administration, hepatitis status, treatment, and comorbidities that might influence survival. The large patient cohort made available by this database affords sufficient statistical power to this investigation.

## Results

The demographic data for the patient cohort are presented in Table 1. A total of 4763 HCC patients were identified in our database, including 3771 men (79.17%) and 992 women (20.83%). The median follow-up time for the cohort was 4.93 months (range 0.03–12). The cohort included 2209 (46.38%) HBV carriers, 812 (17.05%) HCV carriers, and 161 patients infected with both (3.38%). Second to virus infection status, liver cirrhosis (66.02%) was the most common medical condition, followed by diabetes mellitus (34.22%)^17^. Moreover, 1574 (66.41%) and 786 (80.78%) HCC patients had cirrhosis with underlying HBV and HCV, respectively. Sorafenib was administered within 3 months of HCC diagnosis to the majority of patients (51.94%). The duration of sorafenib treatment was less than 2 months in 54.80% of the patients, indicating that more than half of the patients had a poor treatment response and/or tolerance and were not eligible for further sorafenib administration. The majority of the patients in the cohort, received sorafenib alone (n = 4107; 86.23%), while 656 patients (13.77%) received sorafenib and radiotherapy. Patients who received both sorafenib and radiotherapy were more likely to be < 65 years of age, male, without liver cirrhosis or diabetes mellitus. In addition, they were more likely to be HBV-positive, receiving additional therapy such as transarterial chemoembolization.

**Table 1:** Demographic information of advanced hepatocellular carcinoma patients receiving sorafenib combined with radiotherapy or not, n = 4763.

Characteristics	RT, no		RT, yes		*P* value
	N	%	N	%	
Overall patients	4107	86.23	656	13.77	
**Age groups**					< 0.0001
< 35	71	1.73	20	3.05	
35–50	550	13.39	142	21.65	
50–65	1751	42.63	306	46.65	
65 ≥	1735	42.24	188	28.66	
Gender, male	3207	78.09	564	85.98	< 0.0001
**HCC diagnosed to start sorafenib (months)**					< 0.0001
< 3	1912	46.55	562	85.67	
3–6	490	11.93	48	7.32	
6–12	602	14.66	34	5.18	
12≧	1103	26.86	12	1.83	
**Duration of sorafenib using (months)**					< 0.0001
≦2	2390	58.19	220	33.54	
2–4	742	18.07	147	22.41	
4–6	294	7.16	85	12.96	
6 >	681	16.58	204	31.10	
**Prescribed sorafenib dose, (mg/day)**					< 0.0001
200	686	16.70	133	20.27	
400	1369	33.33	161	24.54	
600	199	4.85	28	4.27	
800	1853	45.12	334	50.91	
**Hepatitis virus status**					
Only HBV	1860	45.29	349	53.20	0.0004
Only HCV	730	17.77	82	12.50	
Both HBV/HCV	139	3.38	22	3.35	
Without HBV/HCV	1378	33.55	203	30.95	
**Other comorbidities**					
Liver cirrhosis	2753	67.03	392	59.76	0.0003
Diabetes mellitus	1428	34.77	202	30.79	0.0462
**Additional therapy after sorafenib**					
TACE	802	19.53	230	35.06	< 0.0001
RFA	164	3.99	27	4.12	0.8818
Hepatectomy	28	0.68	13	1.98	0.0008
Distant metastasis	654	17.05	214	32.72	< 0.0001

*RT* radiotherapy, *HCC* hepatocellular carcinoma, *HBV* hepatitis B infection, *HCV* hepatitis C infection, *TACE* transarterial chemoembolization, *RFA* radiofrequency ablation.

(TACE), or hepatectomy, receiving a standard daily dose of sorafenib with a good response, and have distant metastasis (all *P* < 0.05). However, radiofrequency ablation (RFA) revealed no significant difference between the groups. The total radiation dose (Q1–Q3) ranged from 40–55 Gy (median, 50 Gy) in 15–28 fractions (median, 25 fractions).

**Figure 1:**
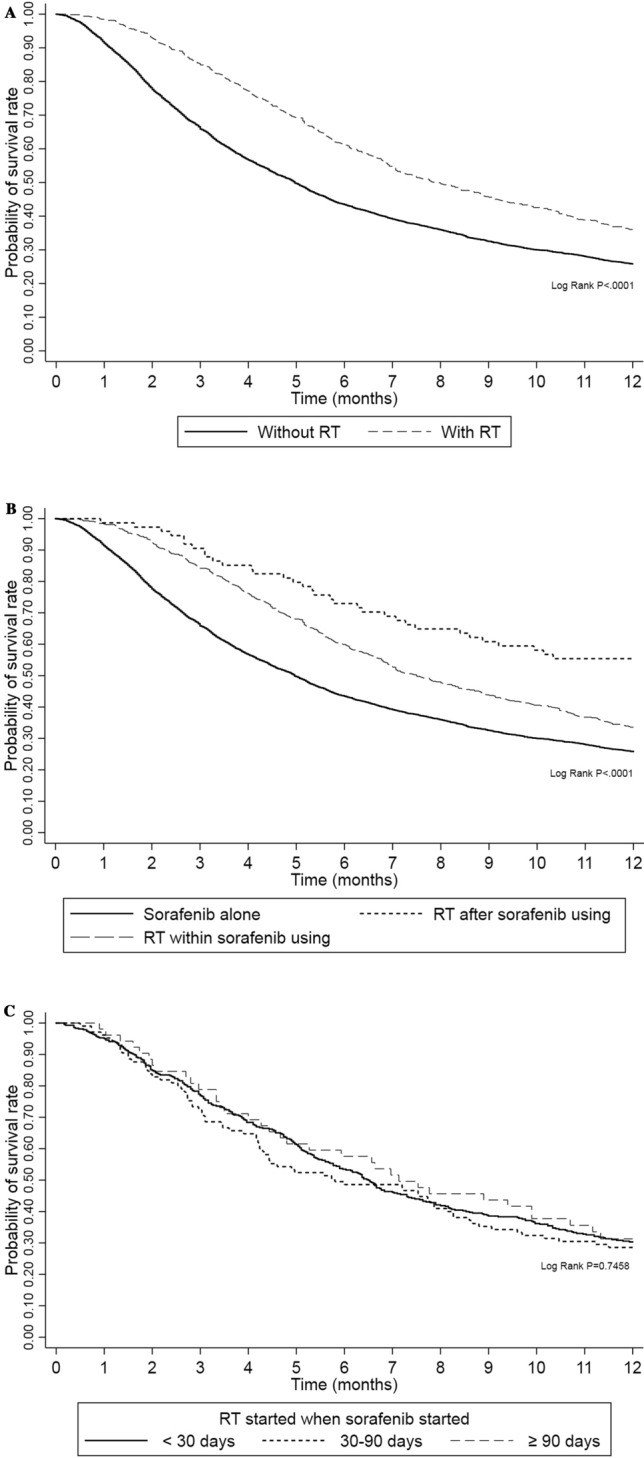
(**A**) The 1-year survival rate in advanced hepatocellular carcinoma patients receiving sorafenib combined with radiotherapy (RT) or not. (**B**) The 1-year survival rate in advanced hepatocellular carcinoma patients between different therapy groups: sorafenib alone, radiotherapy (RT) within or after sorafenib using. (**C)** The 1-year survival rate in advanced hepatocellular carcinoma patients receiving sorafenib according to different radiotherapy (RT) timing.

**Figure 2:**
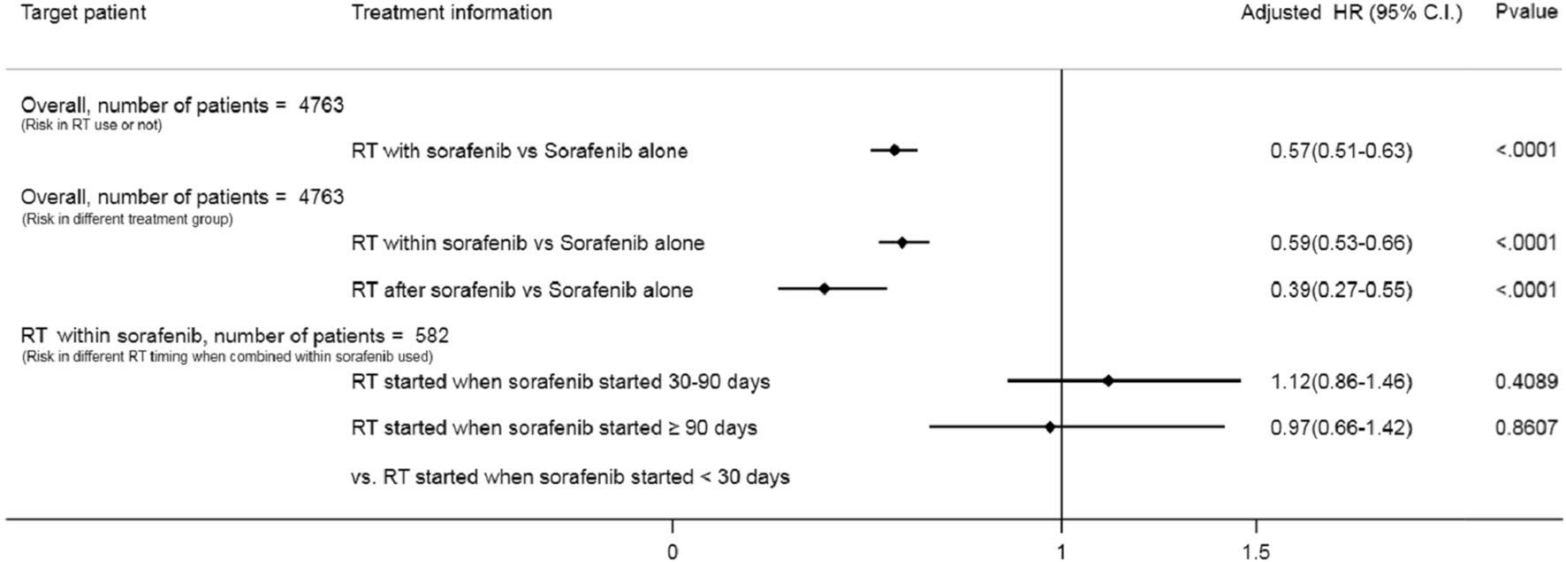
Risk of 1-year mortality of advanced hepatocellular carcinoma patients receiving sorafenib between radiotherapy (RT) use and different timing.

For the total patient cohort, the median OS was 5.33 months (95% CI 5.17–5.57). As shown in Fig. 1A, patients receiving radiotherapy had a higher 1-year survival rate than did those without radiotherapy (*P* < 0.001). The results of uni- and multivariate analysis indicate that after adjusting for various confounders, the increased survival by radiotherapy remained (adjusted hazard ratio [aHR] 0.57; 95% CI 0.51–0.63) (Fig. 2). Regarding the timing of radiotherapy, 582 patients (12.22%) received concurrent sorafenib and radiotherapy, and 74 patients (1.55%) received radiotherapy after sorafenib failure (Supplementary Table 1). Uni- and multivariate analysis revealed that HCC patients receiving radiotherapy after sorafenib had higher 1-year survival (aHR, 0.39; 95% CI 0.27–0.54) than did radiotherapy within sorafenib or sorafenib alone (Figs. 1B, 2). To determine whether the timing of radiotherapy influences its beneficial effect within sorafenib use, we compared the 1-year survival between patients with different radiotherapy start times relative to the start of sorafenib use (< 30, 30–90, and > 90 days). We observed that the difference between these 3 groups was not statistically significant (Figs. 1C, 2).

## Discussion

In this large-scale analysis of a national cancer registry database, our results indicate that patients treated with sorafenib plus radiotherapy had higher 1-year survival than did those treated with sorafenib alone. Multivariate analysis also indicated that sorafenib plus radiotherapy increased survival after adjusting for confounders. Further stratified analysis of the effect of radiotherapy timing revealed that radiotherapy given after sorafenib treatment resulted in better 1-year survival than did radiotherapy within sorafenib use or sorafenib alone.

Compared to previous studies, this investigation has several strengths. First, to the best of our knowledge, this is the first nationwide study to evaluate the efficacy of sorafenib with or without combination radiotherapy in advanced HCC. Our large cohort (n = 4763) and complete follow‐up support the credibility of our results. Second, the use of national databases (TCR and NHIRD), which provide comprehensive data regarding cancer treatment in Taiwan, we were able to establish inclusive patient information including comorbidities, dosage and duration of sorafenib treatment, and radiotherapy treatment. We could perform an in-depth assessment of the effect of these factors on survival. Finally, the use of stratified analysis allowed us to investigate the effect of radiotherapy timing when combined with sorafenib, providing useful information to assist physicians in choosing a treatment strategy.

Several previous studies assessing the feasibility and benefit of sorafenib with radiotherapy for treating advanced HCC report promising early results^11,13^. Cha et al. reported that this combined treatment provided a complete in-field response rate, markedly decreasedα-fetoprotein, and acceptable treatment-related toxicities^11^. Chen et al. report that of 33 patients treated with radiotherapy with concurrent and sequential sorafenib, 22 (55.0%) had achieved complete or partial remission at the initial assessment^18^. Wada et al. also reported that advanced HCC patients receiving sorafenib in combination with radiotherapy had a longer survival than did those treated with sorafenib alone (31.2 vs. 12.1 months), and severe adverse events were comparable among these two groups^13^. In a single arm, phase 2 prospective trial of concurrent chemoradiotherapy followed by sequential sorafenib, Kim et al. reported favorable survival with tolerable toxicity^19^. In addition to these clinical studies, many preclinical studies have proven that combined sorafenib and radiotherapy has a synergistic effect^7,16,20^. Yu et al. found that sorafenib strengthened the response to radiation by inhibiting DNA repair and tumor angiogenesis^16^. Radiation therapy is reported to regulate the expression of many apoptotic and anti-apoptotic genes by activating the NF-κB signaling pathway, altering tumor immunogenicity^21^. Consistent with these studies, our results showed that the addition of radiotherapy to sorafenib treatment in advanced HCC patients increased the 1-year OS over that of sorafenib alone. Together, this evidence indicates that sorafenib in combination with radiotherapy is a viable therapeutic option for advanced HCC patients.

Although the use of sorafenib in combination with radiotherapy for advanced HCC is widely described, the optimal timing of radiotherapy relative to sorafenib treatment remains controversial^15,16^. Plastaras et al. reported that sorafenib increases the efficacy of radiation treatment by blocking Raf/MAPK and VEGFR pathways, and radiotherapy followed by sorafenib was associated with the greatest delay in tumor growth^22^. Wachsberger et al. reported that antiangiogenic agents may inhibit tumor hypoxia and prevent revascularization when combined with sequential radiotherapy^23^. In this study, we found that HCC patients receiving radiotherapy after sorafenib use had a higher 1-year OS than did those receiving radiotherapy within sorafenib use or sorefenib alone. A possible explanation for these findings is that concurrent radiotherapy with sorafenib has an intolerable toxicity profile, which has been reported to decrease the compliance rate and lead to a poorer prognosis among patients experiencing severe toxicity^24,25^. Consistent with these reports, a phase I study of concurrent stereotactic body radiation therapy and sorafenib reported that irradiation of a greater effective liver volume together with sorafenib administration led to intolerable luminal GI toxicity, including bowel bleeding and obstruction^26^. Second, as shown in Table 1, our results indicate that few patients with advanced HCC had an initial response to sorafenib treatment, and only 18.5% were able to stay on the treatment for more than 6 months. These findings suggest that radiotherapy might improve the survival of advanced HCC patients who do not respond to sorafenib. For example, radiotherapy targeting portal vein tumor thrombosis and locoregional therapy for intrahepatic HCC could be promising treatment strategies for these patients^9^.

Notably, all patients in our study were treated with sorafenib first, with or without radiotherapy, because sorafenib is currently recognized as the standard therapy and is thus required for coverage by the National Health Insurance. This insurance requirement remains, even though sorafenib and radiation are proven to have synergistic antitumor effects^11,13,16^. Some studies also reported that sequential treatment with radiation followed by sorafenib appears to be more efficacious against HCC both in vitro and in vivo than either agent given alone or concurrently^15^. In this study, we could not differentiate the actual clinical effect between patients treated by concurrent sorafenib with radiotherapy, radiotherapy first followed by sorafenib, and sorafenib first followed by concurrent sorafenib with radiation. Thus, the radiotherapy within sorafenib group was divided into three subgroups according to different timing relative to the start of sorafenib (after sorafenib start, < 30, 30–90 and > 90 days). However, no significant difference was observed in 1-year survival between these three subgroups (Figs. 1C, 2). Further studies are needed to determine the optimal timing of radiation therapy in this concurrent treatment.

This study has several limitations. First, the administration of sorafenib without interruption or dose reduction was difficult due to its unacceptable treatment-related toxicity. Because information regarding sorafenib- and radiation-related toxicity was not available in our database, we could not assess the compliance rate of sorafenib use in these patients. We assumed that all medications were taken by the patients as prescribed until tumor progression; therefore, the sorafenib dosage may be overestimated^25^. Second, although modern radiotherapy such as stereotactic body radiation therapy (SBRT) and proton therapy provide better tumor coverage and sparing of normal tissues, our database did not provide enough information regarding these techniques^27^. However, the effect of these modern radiotherapy on survival might be greater than that observed in our analysis. Further research comparing the survival benefits of different radiation techniques is needed to clarify the details of this association. Third, the patient cohort was 99% Taiwan residents, most of whom are Asian; racial variations are known to affect the etiology of HCC. For example, HCC is commonly associated with HBV infection in Asia, with HCV infection in Japan and Western countries, and with alcoholism in Western countries. Our results should be further confirmed in patients in other geographic regions due to variations in the efficacy of sorafenib between races^28^. Finally, although a recent randomized, phase III trial (IMbrave150) revealed that advanced HCC patients receiving combination therapy with the antiangiogenic agent bevacizumab plus the immune checkpoint inhibitor atezolizumab had greater survival than did those treated with conventional sorafenib monotherapy, radiotherapy should be considered an option for treating this challenging disease^29,30^.

In conclusion, our results establish a clear association between radiotherapy and improved outcomes of sorafenib treatment in advanced HCC patients. Prospective studies of combined radiotherapy and sorafenib in HCC patients are needed to confirm our findings.

## Methods

### Data sources

Our nationwide cohort analysis used the Taiwan Cancer Registry (TCR) and National Health Insurance Research database (NHIRD) to identify HCC diagnosis, sorafenib administration, and radiation therapy^3,4^. The TCR database captures 97% of the cancer cases in Taiwan and presents excellent data quality compared to other well-established cancer registries^31–33^. The study protocol was approved by the Ethics Committee of the Institutional Review Board of Chi-Mei Medical Center in Taiwan (IRB: 10905-E03).

### Study population

The International Classification of Diseases, Ninth Revision, Clinical Modification (ICD-9-CM) code 155.0 was used to identify patients diagnosed with HCC from January 2012 to December 2015. The follow-up period began on the diagnosis date of HCC and ended on December 31, 2016. Data regarding hepatitis B virus (HBV) or hepatitis C virus (HCV) infection were obtained for the period from 12 months before until 12 months after HCC diagnosis. Comorbidities based on ICD-9-CM codes included HBV (070.20, 070.22, 070.30, 070.32), HCV (070.41, 070.44, 070.51, 070.70, 070.71), liver cirrhosis (571), and diabetes mellitus (250)^34,35^. Patients with a history of cancer, a lack of clear demographic or tumor information, aged < 18 years, and a history of previous systemic therapy were excluded. Patients administered sorafenib were reimbursed without co-payment by the National Health Insurance (NHI) if meeting the Barcelona Clinic Liver Cancer (BCLC) criteria for advanced stage HCC that was not amenable to either surgical resection or locoregional therapy and exhibiting a liver functional reserve of Child–Pugh class A. Sorafenib was administered at a dosage of 400 mg twice a day for 2 months and was re-evaluated every 2 months to approve the next term of use via imaging evidence showing no disease progression^3,4^.

### Study variables and measurements

Demographic data including age, sex, comorbid conditions, hepatitis B or C virus status, and the use of sorafenib, radiotherapy, and additional locoregional therapy were also analyzed. The main endpoint was 1-year survival. Deaths from cancer and other conditions were extracted from the TCR database.

### Statistical analysis

The distribution difference between HCC patients treated with and without radiotherapy was estimated using Pearson’s chi-square test for categorical variables and the Wilcoxon ranked sum test for continuous variables. The Kaplan–Meier plot was used to present the overall survival (OS) curve with the log-rank test for comparison. The risk of mortality was presented as the hazard ratios (HR) with 95% confidence interval (CI) and calculated using the Cox proportional hazard model for all selected risk factors. Based on the scaled Schoenfeld residuals test, the assessment of proportional hazards assumption was approved. Further stratified analysis was used to determine whether radiotherapy had a survival benefit among patients receiving sorafenib alone, radiotherapy within, or after sorafenib use. All statistical analyses were performed using SAS 9.4 for Windows (SAS Institute, Inc., Cary, NC, USA), and Kaplan–Meier curves were plotted using STATA (version 12; Stata Corp., College Station, TX, USA). *P* < 0.05 was considered significant.

## Supplementary Information

Supplementary Table 1.

## Abbreviations

HCC: Hepatocellular carcinoma
TACE: Transarterial chemoembolization
RFA: Radiofrequency ablation
TCR: Taiwan Cancer Registry
NHIRD: National Health Insurance Research database
NHI: National Health Insurance
ICD-9-CM: International Classification of Diseases, Ninth Revision, Clinical Modification
AJCC: American Joint Committee on Cancer
BCLC: Barcelona Clinic Liver Cancer
HBV: Hepatitis B virus
HCV: Hepatitis C virus
OS: Overall survival
HR: Hazard ratio
CI: Confidence interval
SBRT: Stereotactic body radiation therapy
