# Supplementary material for: Effect of radiotherapy on survival in advanced hepatocellular carcinoma patients treated with sorafenib: a nationwide cancer-registry-based study
Source: Sci Rep. 2021 Jan 15;11:1614. doi: 10.1038/s41598-021-81176-w (PMC7810734; doi:10.1038/s41598-021-81176-w)
Supplement: Supplementary file 1 — Supplementary Table 1. [file 41598_2021_81176_MOESM1_ESM.docx]

Supplementary table 1. Demographic information of advanced hepatocellular carcinoma patients between different therapy groups (sorafenib alone, radiotherapy within or after sorafenib using), n= 4763.

| Characteristics | RT, no | | After sorafenib RT | | Within sorafenib RT | | P-value |
| --- | --- | --- | --- | --- | --- | --- | --- |
|  | N | % | N | % | N | % |  |
| Overall patients | 4107 | 86.23 | 74 | 1.55 | 582 | 12.22 |  |
| Age groups |  |  |  |  |  |  | <0.0001 |
| <35 | 71 | 1.73 | 3 | 4.05 | 17 | 2.92 |  |
| 35-50 | 550 | 13.39 | 14 | 18.92 | 128 | 21.99 |  |
| 50-65 | 1751 | 42.63 | 38 | 51.35 | 268 | 46.05 |  |
| 65>= | 1735 | 42.24 | 19 | 25.68 | 169 | 29.04 |  |
| Gender, male | 3207 | 78.09 | 65 | 87.84 | 499 | 85.74 | <0.0001 |
| HCC diagnosed to start sorafenib (months) |  |  |  |  |  |  | <0.0001 |
| <3 | 1912 | 46.55 | 46 | 62.16 | 516 | 88.66 |  |
| 3-6 | 490 | 11.93 | 11 | 14.86 | 37 | 6.36 |  |
| 6-12 | 602 | 14.66 | 12 | 16.22 | 22 | 3.78 |  |
| 12≧ | 1103 | 26.86 | 5 | 6.76 | 7 | 1.20 |  |
| Duration of sorafenib using (months) |  |  |  |  |  |  | <0.0001 |
| ≦2 | 2390 | 58.19 | 40 | 54.05 | 180 | 30.93 |  |
| 2-4 | 742 | 18.07 | 18 | 24.32 | 129 | 22.16 |  |
| 4-6 | 294 | 7.16 | 3 | 4.05 | 82 | 14.09 |  |
| 6> | 681 | 16.58 | 13 | 17.57 | 191 | 32.82 |  |
| Prescribed sorafenib dose, (mg/day) |  |  |  |  |  |  | <0.0001 |
| 200 | 686 | 16.70 | 8 | 10.81 | 125 | 21.48 |  |
| 400 | 1369 | 33.33 | 15 | 20.27 | 146 | 25.09 |  |
| 600≧ | 2052 | 49.97 | 51 | 68.92 | 311 | 53.43 |  |
| Comorbidities |  |  |  |  |  |  |  |
| Liver cirrhosis | 2753 | 67.03 | 45 | 60.81 | 347 | 59.62 | 0.0012 |
| Diabetes mellitus | 1428 | 34.77 | 24 | 32.43 | 178 | 30.58 | 0.1304 |
| HBV | 1999 | 48.67 | 41 | 55.41 | 330 | 56.70 | 0.0009 |
| HCV | 869 | 21.16 | 6 | 8.11 | 98 | 16.84 | 0.0016 |
| Additional therapy after sorafenib |  |  |  |  |  |  |  |
| TACE | 802 | 19.53 | 29 | 39.19 | 201 | 34.54 | <0.0001 |
| RFA | 164 | 3.99 | 3 | 4.05 | 24 | 4.12 | 0.9886 |
| Hepatectomy | 28 | 0.68 | 3 | 4.05 | 10 | 1.72 | 0.0005 |
| Distant Metastasis | 654 | 17.05 | 24 | 32.43 | 190 | 32.76 | <0.0001 |

Abbreviations: RT, radiotherapy; HCC, hepatocellular carcinoma; HBV, hepatitis B infection; HCV, hepatitis C infection; TACE, transarterial chemoembolization; RFA, radiofrequency ablation
